# Consensus statement from the 2014 International Microdialysis Forum

**DOI:** 10.1007/s00134-015-3930-y

**Published:** 2015-07-21

**Authors:** Peter J. Hutchinson, Ibrahim Jalloh, Adel Helmy, Keri L. H. Carpenter, Elham Rostami, Bo-Michael Bellander, Martyn G. Boutelle, Jeff W. Chen, Jan Claassen, Claire Dahyot-Fizelier, Per Enblad, Clare N. Gallagher, Raimund Helbok, Lars Hillered, Peter D. Le Roux, Sandra Magnoni, Halinder S. Mangat, David K. Menon, Carl-Henrik Nordström, Kristine H. O’Phelan, Mauro Oddo, Jon Perez Barcena, Claudia Robertson, Elisabeth Ronne-Engström, Juan Sahuquillo, Martin Smith, Nino Stocchetti, Antonio Belli, T. Adrian Carpenter, Jonathan P. Coles, Marek Czosnyka, Nil Dizdar, J. Clay Goodman, Arun K. Gupta, Troels H. Nielsen, Niklas Marklund, Ambroise Montcriol, Mark T. O’Connell, Maria A. Poca, Asita Sarrafzadeh, Richard J. Shannon, Jane Skjøth-Rasmussen, Peter Smielewski, John F. Stover, Ivan Timofeev, Paul Vespa, Elizabeth Zavala, Urban Ungerstedt

**Affiliations:** Division of Neurosurgery, Department of Clinical Neurosciences, University of Cambridge, Box 167, Cambridge Biomedical Campus, Cambridge, CB2 0QQ UK; Participants of the 2014 International Microdialysis Forum, University of Cambridge, Cambridge, UK

**Keywords:** Brain chemistry, Microdialysis, Outcome, Subarachnoid hemorrhage, Traumatic brain injury

## Abstract

Microdialysis enables the chemistry of the extracellular interstitial space to be monitored. Use of this technique in patients with acute brain injury has increased our understanding of the pathophysiology of several acute neurological disorders. In 2004, a consensus document on the clinical application of cerebral microdialysis was published. Since then, there have been significant advances in the clinical use of microdialysis in neurocritical care. The objective of this review is to report on the International Microdialysis Forum held in Cambridge, UK, in April 2014 and to produce a revised and updated consensus statement about its clinical use including technique, data interpretation, relationship with outcome, role in guiding therapy in neurocritical care and research applications.

**Electronic supplementary material:**

The online version of this article (doi:10.1007/s00134-015-3930-y) contains supplementary material, which is available to authorized users.

## Introduction

Microdialysis is unique in that it allows the chemistry of the extracellular interstitial fluid to be monitored continuously. Since its conception by Ungerstedt and Pycock in the 1970s [1] and its introduction into clinical practice approximately 25 years ago [2], it has been applied to study the tissue chemistry of several human organs. Most experience has been acquired in the setting of neurocritical care. In this arena, microdialysis has been applied to patients with several conditions, and in particular traumatic brain injury (TBI) and subarachnoid hemorrhage (SAH). There is no doubt that this technique has increased our understanding of the pathophysiology of these disease processes [3]. Furthermore, microdialysis has evolved into a clinical tool for the management of patients on an individual intention-to-treat basis.

In neurocritical care, microdialysis data is typically collected together with intracranial pressure (ICP) [allowing calculation of cerebral perfusion pressure (CPP)] and brain tissue oxygen tension (PbtO_2_). Microdialysis complements these techniques by providing additional information on substrate delivery and metabolism at the cellular level. It thus provides the most direct means to monitor the fundamental process of “energy failure”. Of critical importance is that such measurements can be made in real time at the bedside.

In 2003, a group of experts met to review the status of microdialysis as a clinical monitor. This culminated in the publication of a consensus statement in 2004 [4] providing guidance on the use of the technique in TBI and SAH patients. More recently, the role of microdialysis has been evaluated by participants of the International Multidisciplinary Consensus Conference on Multimodality Monitoring [5].

In April 2014, an international forum was convened in Cambridge, UK, with the aim of reviewing evidence for the clinical application of microdialysis in neurocritical care and producing a revised and updated consensus statement [4]. Since the original consensus statement, ~680 articles have been published on microdialysis in neurocritical care. With this increased experience, there was a need to update the 2004 consensus statement. Although there was some overlap between the objectives of this meeting and that of the International Multidisciplinary Consensus Conference on Multimodality Monitoring, i.e. to review the evidence for using microdialysis to guide clinical care, the principal objective of the International Forum in Microdialysis differed in that we aimed to combine literature review with expert opinion to produce practical guidance for the use of cerebral microdialysis as a clinical monitor and to help guide future clinical studies utilizing cerebral microdialysis.

## Methods

The senior authors selected specific ‘key speakers’ to review a particular area of the literature. These individuals were selected based on their experience and contribution to the literature on a particular aspect of microdialysis monitoring. See Appendix 1 in the supplementary material for a list of key speakers and for the topics they each reviewed. The other participants of the meeting were identified through literature review and by correspondence with the key speakers who were able to identify other clinicians and scientists active in using microdialysis in neurocritical care patients. At the meeting, the literature was presented to the whole group followed by discussion to allow consensus generation. After the meeting, the recommendations were circulated to all participants allowing further discussion and revision.

In addition, for the purposes of the consensus statement, we performed a PubMed database search using the term microdialysis plus one of the following terms: ‘traumatic brain injury’, ‘brain injury’, ‘trauma’, ‘subarachnoid hemorrhage’, ‘stroke’, ‘epilepsy’, ‘intracerebral hematoma’ and ‘cost effectiveness’. We restricted our review to using articles published in the English language. Where recommendations are based on published observational data, the relevant references are given although formal grading was not performed. Where references are not provided, the recommendations are based on expert opinion.

## Discussion

### Advances since the 2004 consensus statement

Over the past 10 years, there have been significant advances in the clinical utility of microdialysis in neurocritical care. Evidence from large numbers of patients on how brain chemistry relates to clinical outcome means that we can better define pathological thresholds for microdialysis values. In addition, there is increasing evidence of how different therapeutic manoeuvres can improve chemistry. For a summary of the main advances since the 2004 consensus statement, please see Table 1.

**Table 1:** Summary of advances since the consensus statement by [4]

	2004 consensus statement [4]	Current consensus statement
Microdialysis methodology	Monitoring of small molecules using standard 10-mm 20-kDa catheter	Advances in monitoring of large molecules, with experience of using 100-kDa membrane and colloid for perfusate [13–20]
	Focus on microdialysis metabolites as a marker of ischemia and cell damage	Novel applications of microdialysis for monitoring and understanding brain pathology following TBI and SAH
Core data reporting information	Not defined	Details are given of the essential information required to interpret and compare microdialysis data
Reference values	Not defined	Pathological thresholds defined for glucose, lactate and the LP ratio [6, 51, 53–55, 68–73, 79, 80]
Tiered approach to microdialysis metabolites for clinical application	Not defined	Glucose and LP ratio more clinically useful than glutamate and glycerol in TBI and SAH patients
Guidance for microdialysis-directed management	Not given	Suggested therapeutic interventions for when glucose is low (<0.2 mM) and for when the LP ratio indicates ischemia ± tissue hypoxia
Monitoring in TBI	Guidance on catheter placement in focal or diffuse injury	Guidance on single or multiple catheter placement based on whether the injury is focal or diffuse and based on the aims of microdialysis monitoring
Monitoring in SAH	Guidance on catheter placement in the tissue at risk	Two principal indications for microdialysis monitoring are defined:
		1. As a primary monitoring device in mechanically ventilated patients
		2. As a monitor of patients with a secondary neurological deterioration

Most attention has been directed at the clinical utility to monitor TBI and SAH patients: see Table 2 for a summary of how brain chemistry relates to different aspects of the care of patients with TBI and SAH. Microdialysis has also been used in other neurological conditions including intracerebral hemorrhage [6], acute ischemic stroke [7–9], hepatic encephalopathy [10] and epilepsy [11, 12]. However, there is insufficient evidence at present to specifically incorporate the application of microdialysis in these conditions into the consensus statement.

**Table 2:** Summary of the evidence for how brain chemistry relates to different aspects of the management of patients with TBI and SAH

How microdialysis monitoring can be used in neurocritical care	Traumatic brain injury	Subarachnoid hemorrhage
Outcome and prognostication	[51, 53, 78]	[67, 79, 81]
Early warning system of secondary insults	[26, 27]	[28, 29, 80, 82]
Monitoring and treatment of low cerebral glucose; guiding systemic glucose management and insulin use	[56, 61, 62, 64, 65]	[56, 63, 83, 84]
Monitoring during CPP-augmentation/reduction	[48, 85, 86]	[54, 87]
Monitoring during neurological wake-up test (tolerating moderate rises in ICP)	[25, 88]	
Deciding on transfusion thresholds		[89]
Evaluating the effect of body temperature on cerebral chemistry	[90]	[91]
Monitoring after decompressive craniectomy	[92]	[93]

In addition to recent advances as a clinical monitor, microdialysis continues to be a powerful research tool with numerous, varied and several novel applications that provide insight into various aspects of cerebral biology and pathophysiology. For a summary of on-going and future research, see Table 3. Overall, further research should be directed at the integration of brain chemistry and other clinical monitoring data to better define targets for the individualized goal-directed management of the brain-injured patient.

**Table 3:** A summary of on-going microdialysis research applications

Investigating the concept of lactate as a substrate as opposed to a metabolic by-product in select patients	[79, 94]
Use of 100-kDa microdialysis membranes to measure larger molecules including cytokines	[15, 16, 18, 19, 95]
Use of ^13^C-labelled substrates to interrogate metabolic pathways in more detail, e.g., the fate of glucose metabolism (glycolysis vs. pentose phosphate pathway) and the fate of lactate as a substrate	[94, 96, 97]
Monitoring drug penetration across the blood–brain barrier and the effect of drugs on brain chemistry	[98, 99]
Clinical use in pediatric practice	[100–102]
Monitoring of the ionic component of the interstitial space	[103]
Monitoring of biomarkers	[18, 19, 104–111]
Development of microfluidic based on-line assays that give continuous neurochemical information in real time	[23, 24, 112]

### Advances in microdialysis methodology

The technique of microdialysis is well established. For details on technique and on the factors that affect relative recovery, i.e. how the substance measured in the dialysate is related to the free concentration in the tissue interstitial space, please see supplementary material.

Microdialysis is used clinically to estimate extracellular interstitial concentrations of small molecules, but can also be used to recover much larger molecules such as inflammatory mediators from the interstitial fluid. Instead of the standard 20-kDa nominal molecular weight cut-off membrane, which recovers glucose, pyruvate, lactate, glycerol, glutamate, and other small hydrophilic molecules, a 100-kDa membrane is used to also recover larger molecules including cytokines. The recovery of small molecules does not differ between the two membrane types [13]. Increased experience in using microdialysis for large molecules less than 100 kDa has been achieved in the past 10 years. Importantly, the use of colloid in the perfusate (e.g., albumin or dextran) significantly improves the relative recovery of these large molecules [14–16]. However, in some situations, colloid perfusate can cause net influx of fluid into the catheter potentially dehydrating the interstitial space, and dextrans of molecular weight 40–250 kDa may leak through the microdialysis membrane potentially disturbing the interstitial microenvironment [14, 15, 17]. These problems may be overcome by using higher molecular weight dextrans, such as 500-kDa dextran, as colloid in the perfusate [18–20]. A useful alternative colloid to dextran is human serum albumin (HAS), which has been shown to improve recovery for the majority of cytokines compared to crystalloid perfusate without significantly dehydrating the interstitial space [16].

Most experience of microdialysis in neurocritical care has been obtained with hourly measurements although more frequent sampling is possible [21–25]. Hourly sampling appears sufficient to detect the metabolic changes that can sometimes precede episodes of intracranial hypertension in TBI and symptomatic delayed ischemia in SAH [26–29]. Hence, microdialysis has the potential to be used as an early warning system of secondary insults. However, dynamic changes in brain chemistry, for example due to spreading depolarization [21–23] or observed during aneurysm surgery [24, 30], may not be detected with hourly measurements, so there is potentially scope for improved technology with more frequent microdialysis readings in future, which may lead to better warning of adverse events.

### Clinical application in intensive care

The clinical application of microdialysis in neurocritical care has focused on the delivery of glucose and its metabolism via glycolysis to pyruvate, which under oxidative conditions feeds into the tricarboxylic acid (TCA) cycle. Under hypoxic conditions, or if mitochondrial function is compromised, pyruvate is metabolized to lactate. Hence, the LP ratio is used as a marker of aerobic versus “anaerobic” metabolism not requiring oxygen [31, 32]. Glutamate is measured as a marker of hypoxia/ischemia and has been considered as an indicator of excitotoxicity [31–34]. Glycerol is regarded a marker of hypoxia/ischemia and cell membrane breakdown [32, 35–37].

### Safety profile

The technique of cerebral microdialysis is safe. Several published series of patients studied with microdialysis, which include non-brain-injured patients, have not reported adverse events related to microdialysis catheter insertion [29, 38–40]. Cerebral microdialysis has a safety profile at least equivalent to that of intra-parenchymal pressure sensors owing to the catheter’s greater flexibility and small diameter [41]. In most circumstances when an adverse event occurs, it relates to the insertion technique rather than the catheter itself. Cerebral microdialysis has mostly been used as a tool for observational studies. Further evaluation of microdialysis as a clinical monitor should include assessment of potential harm caused by microdialysis-directed interventions.

### Cost-benefit analysis

No cost effectiveness studies evaluating microdialysis in neurocritical care have been performed. One study compared ICP monitoring alone against multimodal monitoring, which consisted of transcranial Doppler, jugular venous oxygen saturation and/or PbtO_2_ monitoring but not microdialysis [42]. Albeit a small study, it demonstrated that increased upfront costs due to consumables and equipment was offset by better clinical outcomes, which meant that multimodal monitoring was cost effective. In TBI patients, there are indications that aggressive management, which includes invasive monitoring, improves outcomes and is cost effective [43–46]. However, these studies have not examined microdialysis monitoring per se.

## **Recommendations from the 2014 International Forum on Microdialysis––the 2014 consensus statement**

### Methodology

- Catheters should be inserted according to local institutional protocols either by twist drill hole, transcranial bolt, or at craniotomy.
- The first hour of microdialysate collected should not be used for clinical monitoring due to unreliable results caused by insertion trauma and the pump flush sequence.
- To monitor glucose, pyruvate, lactate, glycerol and glutamate catheters with a 20- or 100-kDa cut-off are available (100-kDa catheters are not yet FDA-approved, although they are CE marked for use in Europe).
- A flow rate of 0.3 μL/min with hourly sampling is recommended, which is the flow rate most commonly used in the cerebral microdialysis literature.
- Publication of microdialysis data should include the following information (core data reporting):
  - catheter type
  - catheter location based on post-insertion imaging
  - flow rate
  - membrane length
  - perfusion fluid composition
  - time from ictus to monitoring

### Interpretation of cerebral microdialysis

- Microdialysis monitors substrate delivery and metabolism at the cellular level. Chemistry should be interpreted in the context of the clinical condition of the patient and in conjunction with other monitored parameters including ICP, CPP, PbtO_2_, cerebrovascular pressure reactivity (PRx) and systemic parameters, in order to determine the likely cause of perturbed metabolism. For example, a rise in LP ratio associated with a fall in CPP and loss of cerebrovascular reactivity (i.e., a high PRx) indicates that the likely cause of disordered chemistry is ischemia.
- Microdialysis is a focal technique. The heterogeneity of brain injury means that brain chemistry varies in different regions of the brain. In TBI, peri-lesional brain demonstrates more perturbed chemistry, in particular a higher LP ratio, compared to other areas of brain [47–52]. Therefore, brain chemistry should be interpreted according to catheter location in relation to focal injury based on CT/MRI imaging.

#### Glucose

- Glucose is the main substrate for brain metabolism.
- Periods of low glucose (<0.8 mM) are observed in TBI and SAH.
- Low brain glucose is associated with unfavorable outcome [53–57].
- There is also evidence that high brain glucose is associated with unfavorable outcome indicating that there is an optimal range for brain glucose, although, there is currently insufficient data to define this range [51, 58].
- Serum glucose concentration and glycemic control influence brain glucose although this relationship may be lost in injured brain [56, 59–65].
- Brain glucose can be reduced rapidly by secondary insults such as spreading depolarization [22, 66].

#### Lactate/pyruvate ratio

- A high LP ratio is associated with unfavorable outcome [6, 51, 53, 54, 57, 67–73].
- The LP ratio is a marker of cellular redox status.
- The LP ratio is a quantitative measure (independent of relative recovery).
- An increased LP ratio may result from a failure of oxygen delivery (ischemic hypoxia) or from non-ischemic causes (e.g., mitochondrial dysfunction) [74, 75].
- The absolute lactate and pyruvate concentrations should be considered when interpreting a high LP ratio.
- Ischemia and mitochondrial dysfunction are two ends of a spectrum of factors that increase the LP ratio.
- An increase in the LP ratio in the presence of low pyruvate (and low oxygen) indicates ischemia.
- An increase in LP ratio in the presence of normal or high pyruvate (and normal oxygen) indicates mitochondrial dysfunction.

#### Glutamate

- Glutamate is an excitatory amino acid and neurotransmitter. Excess levels are thought to be an additional injurious mechanism and may exacerbate injury in TBI and SAH.
- Excess glutamate release is observed in ischemia [8, 31, 33, 76] and seizures [11, 12, 76, 77].
- There is a described association between glutamate levels, clinical course and outcome in TBI and SAH [29, 78].
- Measuring cerebral glutamate is an option and may be useful in estimating prognosis.

#### Glycerol

- Glycerol is a marker of cell membrane breakdown. It is a potential marker of oxidative stress.
- Glycerol has limited specificity; brain glycerol concentrations are influenced by systemic concentrations. Systemic glycerol concentrations reflect a stress response and/or administration of glycerol-containing substances.
- There is no definitive evidence of a relationship between glycerol and outcome.
- Cerebral glycerol is an option as a marker of cerebral injury.

### Guidance for use of microdialysis in traumatic brain injury and subarachnoid hemorrhage—catheter location, reference values and interventions

#### Traumatic brain injury

- In diffuse TBI, we recommend placing the catheter in the right (non-dominant) frontal lobe.
- In focal TBI, there are different options for catheter placement that depend on whether the goal is to monitor tissue at risk or normal brain, e.g., to guide systemic glucose treatment.
- Where there is a focal lesion, we recommend, if feasible, catheter placement ipsilateral to the lesion and in radiographically normal brain.
- Multiple catheters are an option in focal TBI.
  - E.g., placed at craniotomy for a focal lesion into peri-lesional brain with a contralateral ‘bolt’ catheter in radiographically normal brain.
  - Stereotactic placement is an option but rarely practical.

#### Subarachnoid hemorrhage

- There are two principal indications for the insertion of microdialysis in SAH patients:
  1. As a primary monitoring device in mechanically ventilated (‘poor-grade’) patients.
  2. As a monitor of patients with a secondary neurological deterioration.
- As a primary monitoring device, we recommend catheter location in the watershed anterior cerebral artery–middle cerebral artery (ACA–MCA) territory (frontal lobe) on the same side as the maximal blood load seen on CT or the ruptured aneurysm. If the blood load is symmetrical, we recommend non-dominant frontal lobe placement.
- In patients with a secondary neurological deterioration, catheter location should be guided by local practice to identify tissue at risk (e.g., CT perfusion scanning or trans-cranial Doppler).
- Multiple catheters are an option in SAH.

#### Reference values and interventions

- It is currently difficult to define absolute normal or abnormal values based on the literature. Different groups have used different threshold values to relate microdialysate values to outcome. Furthermore, some authors have used a combination of values to relate microdialysis to clinical outcomes.
- The trend is as important or possibly more important than point values or threshold values.
- It is important to distinguish between normal values, which have been reported in the awake and anesthetised brain of patients undergoing surgery for benign intracranial lesions, and values that characterize pathophysiological disturbance of brain chemistry.
- We propose the following pathological thresholds (one or two stages), for microdialysis at 0.3 µl/min, based on observational studies that have explored statistical differences in outcomes in relation to thresholds of microdialysate values. Microdialysate values observed beyond these thresholds indicate that the area of brain being monitored is ‘at risk’. We propose clinical interventions that may be appropriate in response to disturbed brain chemistry. Further research is needed to elucidate whether these thresholds can be applied to both peri-lesional and to radiographically normal brain and to identify whether interventions directed by these thresholds improve clinical outcomes.
  - Glucose: <0.2 and <0.8 mmol/L [53–55, 73].
  - If brain glucose is low (<0.2 mM), a trial of increasing serum glucose (by intravenous or enteral administration and/or loosening glycemic control) should be considered. Factors to consider when deciding whether this is an appropriate intervention include baseline serum glucose concentration and whether other parameters indicate cerebral ischemia. If baseline serum glucose concentration is high, further increasing the glucose concentration is likely to increase the risk of both neurological and systemic complications from hyperglycemia. The precise definition of blood sugar thresholds for safety is beyond the scope of this manuscript, but frank hyperglycemia should be avoided. If other parameters, such as the LP ratio and PbtO_2_, indicate ischemia, interventions directed at improving cerebral perfusion should be considered first-line.
  - Lactate: >4 mmol/L [51, 73, 79, 80].
  - Lactate/pyruvate ratio: >25 and >40 [6, 51, 53, 54, 68–73].
  - If the LP ratio indicates ischemia, i.e. an increase in the LP ratio in the presence of low pyruvate, CPP augmentation is a therapeutic option.
  - If the LP ratio is increased in the presence of low brain tissue oxygen, interventions that improve oxygen delivery, such as judiciously increasing the cerebral perfusion pressure, increasing PaCO_2_, increasing inspired concentration of oxygen and/or correcting anemia, should be considered. However, all of these interventions have potential side effects, and the choice of intervention will depend on the pre-intervention levels of any given variable, and a consideration of the side effects of the intervention. Thus, for example, in patients with significant hypocarbia, an increase in PaCO_2_ might be the most appropriate intervention, but may be difficult to achieve due to increases in intracranial pressure.

#### Tiered approach to the clinical value of substances

- Accumulating evidence since the last consensus statement indicates that the value of the metabolites can now be considered in a tiered fashion (tier 1 being most robust and useful) for their clinical application as follows. This hierarchy is based on the larger volume of observational data linking glucose and LP ratio with outcome compared to glutamate and glycerol and on the greater potential for glucose and LP ratio to direct clinical interventions.
- Tier 1: glucose and LP ratio.
- Tier 2: glutamate.
- Tier 3: glycerol.

## Summary and future directions

Cerebral microdialysis is a reliable and safe technique that is used in the clinical management of neurocritical care patients and in particular those with severe TBI or SAH. In addition, there are several research applications that are important for developing our understanding of brain physiology, pathophysiology and drug development. Since the 2004 consensus document, there have been significant advances in our understanding of how microdialysis can be used. There is now evidence from large numbers of patients on how abnormal brain chemistry relates to clinical outcome. The measurement of glucose, lactate and the LP ratio are now considered more useful than glutamate and glycerol. The LP ratio, interpreted in the light of absolute pyruvate concentrations and PbtO_2_, can be used to differentiate ischemic from non-ischemic causes of energy dysfunction. Importantly, there is increasing evidence of how different therapeutic manoeuvres influence brain chemistry. Microdialysis is well placed to help guide the management of patients in an individualized and targeted fashion. For its effective use, microdialysis should be integrated into brain multi-modal monitoring systems and interpreted with knowledge of catheter location and clinical context. Future clinical research should focus on assessing the clinical effectiveness of decision-making based on microdialysis, as part of multi-modality monitoring of acute brain injured patients, and its integration into treatment paradigms in neurocritical care.

## Electronic supplementary material

Supplementary material 1 (PDF 2447 kb)
