# Supplementary material for: Consensus statement from the 2014 International Microdialysis Forum
Source: Intensive Care Med. 2015 Jul 21;41:1517–28. doi: 10.1007/s00134-015-3930-y (PMC4550654; doi:10.1007/s00134-015-3930-y)
Supplement: Supplementary file 1 — Supplementary material 1 (PDF 2447 kb) [file 134_2015_3930_MOESM1_ESM.pdf]

SUPPLEMENTARY MATERIAL

Microdialysis Methodology

The clinical microdialysis catheter is carefully placed within the cerebral parenchyma either via a cranial access device ("bolt") or tunnelled via a twist drill hole or placed under direct vision at craniotomy. The catheter consists of two concentric tubes. The outer tube is connected to a syringe pump that delivers perfusion fluid. This flows through the outer tube down to the tip, where the final 10 mm (typically) of the wall of the tube is made of a semi-permeable dialysis membrane, across which diffusion of molecules takes place bi-directionally between the perfused fluid and the brain interstitial fluid outside the catheter, depending on the current chemical gradient across the dialysis membrane (i.e. the chemical composition of the perfused fluid in relation to the interstitial fluid). The outer tube has a blind end (with a gold tip that is visible on a CT/MRI scan), and the perfusion fluid, now termed microdialysate, returns up the inner tube to be analyzed, typically by collection in a vial changed hourly. The microdialysate thus contains molecules that have diffused into the catheter from the brain interstitial fluid. A schematic diagram of the microdialysis catheter tip is shown in Supplementary Fig. 1.

The concentration of a substance measured in the dialysate is related to the free concentration in the tissue interstitial fluid by the mean relative recovery (the dialysate concentration as a percentage of the true interstitial concentration). This reflects properties of the catheter and perfusion fluid as well as properties of the tissue. Ratios of concentrations are independent of changes in relative recovery and depend less on tissue properties. The best pairings are substances whose concentrations are likely to change in opposite fashions (i.e. one increases while the other decreases) such as the lactate/pyruvate ratio (LP ratio) [1-3].

Perfusion fluid is pumped continuously through the microdialysis catheter, which means that equilibrium across the semi-permeable membrane is never reached. The slower the fluid flows through the catheter, the closer the concentration of the substance measured in the dialysate is to its concentration in the interstitial fluid. Typically, a 10 mm length membrane and a flow rate of 0.3 µL/min is used to monitor the human brain. This equates to a mean relative recovery of approximately 65 to 72 % for the molecules typically measured in the clinical setting [4]. At faster flow rates, recovery declines; at 1 µL/min recovery is between 21 and 34 % for the same molecules [4] although other substances such as neurotransmitters maintain high recovery at faster flow rates. Important tissue properties that can influence the mean relative recovery include tissue edema and tissue damage close to the catheter membrane both of which reduce availability of a substance near the catheter. An unexplored factor is the influence of ICP on relative recovery. Hence, microdialysate values should be presented as the measured concentration, unadjusted for relative recovery determined *in vitro*, and with details of the catheter and perfusion fluid (see core data reporting recommendations).

The choice of perfusion fluid influences the microenvironment and concentration of molecules recovered. Perfusion fluid used commercially for routine clinical monitoring of the brain is an un-buffered solution with an ionic composition similar to that of interstitial fluid. This ensures that there is no significant shift of hydrogen ions, the major cations (sodium, potassium and calcium) or anions (chloride) across the microdialysis membrane. In addition, magnesium (a predominantly intracellular cation) is added to ensure that NMDA glutamate channels do not become activated as a result of magnesium depletion. The influence of changes in the ionic composition on the recovery of molecules is in practice small in the human brain [4].

The bedside analyzers used for measuring the standard metabolites glucose, pyruvate, lactate, glutamate and glycerol are automated enzymatic colorimetric analyzers that are

designed to handle small sample volumes. The only current commercially available clinical microdialysis analyzer is the ISCUSflex (from M Dialysis AB, Stockholm, Sweden); earlier models (ISCUS and CMA600) from the same manufacturer are still in use. Each measurement uses between 0.2 and 1 µL of the sample for analysis depending on the analyte. The leftover microdialysates in the collection vials can then be stored for later off-line analysis of other substances e.g. other amino acids, biomarkers, and drugs (although for drug pharmacokinetic studies it is recommended that in vivo recovery is estimated at the time of sampling). It is recommended to run control samples regularly (at a minimum of two concentration levels) as a complement to the automatic calibrations to ensure proper quality control of the analyser.

Supplementary Figure 1

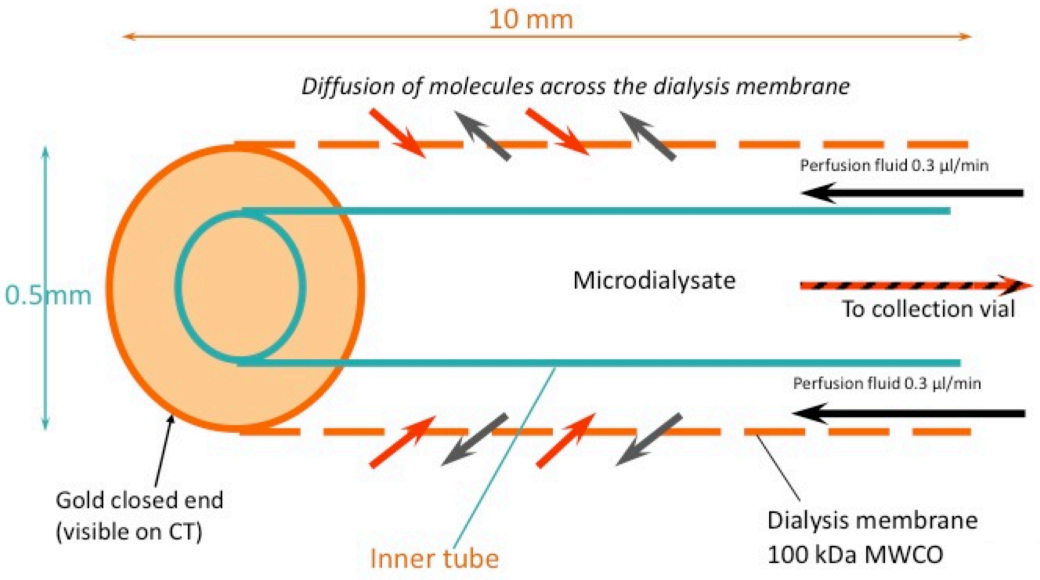

Figure originally published by Shannon RJ et al. in Journal of Pharmacokinetics and Pharmacodynamics 40:343–358 under a Creative Commons Attribution Licence [5].

REFERENCES

1. Persson L, Hillered L (1996) Intracerebral Microdialysis. J Neurosurg 85:984–985. doi: 10.3171/jns.1996.85.5.0984

2. Reinstrup P, Ståhl N, Mellergård P, et al. (2000) Intracerebral microdialysis in clinical practice: baseline values for chemical markers during wakefulness, anesthesia, and neurosurgery. Neurosurgery 47:701–710. doi: 10.1227/00006123-200009000-00035

3. Parkin MC, Hopwood SE, Boutelle MG, Strong AJ (2003) Resolving dynamic changes in brain metabolism using biosensors and on-line microdialysis. TrAC Trends in Analytical Chemistry 22:487–497. doi: 10.1016/S0165-9936(03)00912-9

4. Hutchinson PJ, O'Connell MT, Al-Rawi PG, et al. (2000) Clinical cerebral microdialysis: a methodological study. J Neurosurg 93:37–43. doi: 10.3171/jns.2000.93.1.0037

5. Shannon RJ, Carpenter KLH, Guilfoyle MR, et al. (2013) Cerebral microdialysis in clinical studies of drugs: pharmacokinetic applications. J Pharmacokinet Pharmacodyn 40:343–358. doi: 10.1007/s10928-013-9306-4

APPENDIX 1

A list of speakers and topics discussed at the International Microdialysis Forum

'Normal' brain chemistry	R Helbok
Glucose & hyperglycolysis	D K Menon
Lactate/pyruvate ratio: ischaemia and/or mitochondrial dysfunction	C-H Nordström
Lactate: fuel or waste	M Oddo
Glycerol: disordered energy metabolism with cell membrane breakdown and/or oxidative stress?	L Hillered
Glutamate: a marker of excitotoxicity?	C Robertson
Relationship between MD, tissue oxygen and other parameters	K O'Phelan
Influence of catheter location	P Enblad
Data analysis and reporting	J Classen and U Ungerstedt
Online microdialysis and sensors	M G Boutelle
Microdialysis as a tool to explore the ionic profile of the brain extracellular space	J Sahuquillo
Drug development	C Dahyot-Fizelier
Pediatric applications	C N Gallagher
Monitoring of inflammation	A Helmy
Serum vs. microdialysis biomarkers	B Bellander
13C labelling studies	K L H Carpenter
Relationship between microdialysis and clinical course in stroke and SAH	E Ronne-Engström
Relationship between microdialysis and clinical course in TBI	J Perez Barcena
Microdialysis-guided therapeutic interventions	M Smith and N Stocchetti

APPENDIX 2

A list of all participants and conflicts of interest

Name		Department	COI
Bo-Michael	Bellander	Division of Neurosurgery, Dept. of Clinical Neuroscience, Karolinska Institute, Stockholm, Sweden	Travel grant received from CMA Microdialysis AB to attend an academic meeting.
Antonio	Belli	NIHR Surgical Reconstruction and Microbiology Research Centre, Queen Elizabeth Hospital Birmingham and University of Birmingham, Birmingham, UK	No conflict of interest.
Martyn G	Boutelle	Department of Bioengineering, Imperial College London, London UK	No conflict of interest.
Keri LH	Carpenter	Division of Neurosurgery, Dept. of Clinical Neurosciences, University of Cambridge, Cambridge Biomedical Campus, Cambridge, UK	No conflict of interest.
T Adrian	Carpenter	Wolfson Brain Imaging Centre, Dept. of Clinical Neurosciences, University of Cambridge, Cambridge Biomedical Campus, Cambridge, UK	No conflict of interest.
Jeff W	Chen	Department of Neurosurgery, Legacy Emanuel Medical Center, Portland, OR, USA	No conflict of interest.
Jan	Claassen	Neurological Intensive Care Unit, Columbia University College of Physicians & Surgeons, New York, NY, USA	No conflict of interest.
Jonathan P	Coles	Division of Anaesthesia, Dept. of Medicine, University of Cambridge, Cambridge Biomedical Campus, Cambridge, UK	No conflict of interest.
Marek	Czosnyka	Division of Neurosurgery, Dept. of Clinical Neurosciences, University of Cambridge, Cambridge Biomedical Campus, Cambridge, UK	No conflict of interest.
Claire	Dahyot-Fizelier	Neurological Intensive Care Unit, Division of Anaesthesia and Surgical Intensive Care, Inserm U1070, University Hospital of Poitiers, France.	No conflict of interest.

Nil	Dizdar	Department of Neurology, University Hospital Linköping, Sweden	Microdialysis catheters received from CMA Microdialysis AB for a research study in 2007.
Per	Enblad	Department of Neurosurgery, University Hospital Uppsala, Sweden	No conflict of interest.
Clare N	Gallagher	Division of Neurosurgery, Dept. of Clinical Neurosciences, University of Calgary, Alberta, Canada	No conflict of interest.
J Clay	Goodman	Department of Pathology & Immunology, Baylor College of Medicine, Houston, TX, USA	No conflict of interest.
Arun K	Gupta	Division of Anaesthesia, Dept. of Medicine, University of Cambridge, Cambridge Biomedical Campus, Cambridge, UK	No conflict of interest.
Raimund	Helbok	Neurocritical Care Unit, Department of Neurology, Innsbruck Medical University, Austria	No conflict of interest.
Adel	Helmy	Division of Neurosurgery, Dept. of Clinical Neurosciences, University of Cambridge, Cambridge Biomedical Campus, Cambridge, UK	No conflict of interest.
Lars	Hillered	Department of Neuroscience, Division of Neurosurgery, Uppsala University, Sweden	No conflict of interest.
Peter JA	Hutchinson	Division of Neurosurgery, Dept. of Clinical Neurosciences, University of Cambridge, Cambridge Biomedical Campus, Cambridge, UK	No conflict of interest.
Ibrahim	Jalloh	Division of Neurosurgery, Dept. of Clinical Neurosciences, University of Cambridge, Cambridge Biomedical Campus, Cambridge, UK	No conflict of interest.
Peter D	Le Roux	The Brain and Spine Center, Suite 370, Medical Science Building, Lankenau Medical Center, 100 E. Lancaster Ave Wynnewood PA, USA	Previous research support received from CMA Microdialysis AB.

Sandra	Magnoni	Department of Anesthesiology and Intensive Care<br>Fondazione IRCCS Cà Granda-Ospedale Maggiore Policlinico, Milan, Italy	No conflict of interest.
Halinder S	Mangat	Departments of Neurology and Neurosurgery, Division of Stroke & Critical Care, Weill Cornell Medical College, New York, NY, USA	Travel grant received from M Dialysis to attend the International Microdialysis Forum 2014
Niklas	Marklund	Departments of Neuroscience and Neurosurgery, Uppsala University, Uppsala University Hospital, Uppsala, Sweden.	No conflict of interest.
David K	Menon	Division of Anaesthesia, University of Cambridge, Cambridge Biomedical Campus, Cambridge, UK	No conflict of interest.
Ambroise	Montcriol	Intensive Care Unit, Sainte Anne Military Teaching Hospital, Toulon, France	No conflict of interest.
Troels H	Nielsen	Department of Neurosurgery, Odense University Hospital, Denmark	Current research support from M Dialysis.
Carl-Henrik	Nordström	Department of Neurosurgery, Odense University Hospital, Denmark	No conflict of interest.
Mark T	O'Connell	Probe Scientific Limited, Bedford Technology Park, Thurleigh, Bedford, UK	No conflict of interest.
Kristine H	O'Phelan	Department of Neurology, Miller School of Medicine, University of Miami, Miami, USA	No conflict of interest.
Mauro	Oddo	Department of Intensive Care Medicine, CHUV-University Hospital, Faculty of Biology and Medicine, University of Lausanne, CH-1011 Lausanne, Switzerland	No conflict of interest.
Jon	Perez Barcena	Neurocritical Care Department, Jackson Memorial Hospital, University of Miami, Miami, USA	No conflict of interest.

Maria A	Poca	Department of Neurosurgery, Neurotrauma and Neurosurgery Research Unit, Vall d'Hebron University Hospital, Barcelona, Spain	No conflict of interest.
Claudia	Robertson	Department of Neurosurgery, Baylor College of Medicine, Houston, TX, USA	No conflict of interest.
Elisabeth	Ronne-Engström	Department of Neuroscience, Division of Neurosurgery, Uppsala University, Sweden	No conflict of interest.
Elham	Rostami	Department of Neuroscience, Division of Neurosurgery, Uppsala University, Sweden	No conflict of interest.
Juan	Sahuquillo	Department of Neurosurgery, Vall d'Hebron University Hospital, Universidad Autonoma de Barcelona, Barcelona, Spain	No conflict of interest.
Asita	Sarrafzadeh	Department of Neurosurgery, Heidelberg University Hospital<br>Im Neuenheimer Feld 400<br>69120 Heidelberg<br>Germany	No conflict of interest.
Richard J	Shannon	Division of Neurosurgery, Dept. of Clinical Neurosciences, University of Cambridge, Cambridge Biomedical Campus, Cambridge, UK	No conflict of interest.
Jane	Skjøth-Rasmussen	Departments of Neurosurgery, Odense University Hospital, Odense, Denmark	Accepted invitation to attend a microdialysis course organised by M Dialysis.
Peter	Smielewski	Division of Neurosurgery, Dept. of Clinical Neurosciences, University of Cambridge, Cambridge Biomedical Campus, Cambridge, UK	No conflict of interest.
Martin	Smith	Neurocritical Care Unit, The National Hospital for Neurology and Neurosurgery, UCLH/UCL National Institute for Health Research Biomedical Research Centre, London, UK	No conflict of interest.

Nino	Stocchetti	Milan University, Terapia Intensiva Neuroscienze, Fondazione IRCCS Ca' Granda, Ospedale Maggiore Policlinico, Milan, Italy	No conflict of interest.
John F	Stover	Fresenius Kabi, Bad Homburg, Germany / Surgical Intensive Care Medicine, University Hospital, Zurich, Switzerland	No conflict of interest.
Ivan	Timofeev	Division of Neurosurgery, Dept. of Clinical Neurosciences, University of Cambridge, Cambridge Biomedical Campus, Cambridge, UK	No conflict of interest.
Urban	Ungerstedt	Department of Physiology and Pharmacology, Karolinska Institute, Stockholm, Sweden	No conflict of interest.
Paul	Vespa	UCLA Brain Injury Research Center, David Geffen School of Medicine, University of California, Los Angeles, Los Angeles, California	No conflict of interest.
Elizabeth	Zavala	Surgical ICU, Department of Anesthesiology. Clinic Hospital, IDIBAPS. Universidad de Barcelona, Barcelona, Spain	Technical and teaching support received from CMA Microdialysis AB in 2000.
